# The Ovine Cerebral Venous System: Comparative Anatomy, Visualization, and Implications for Translational Research

**DOI:** 10.1371/journal.pone.0092990

**Published:** 2014-04-15

**Authors:** Anke Hoffmann, Michael H. Stoffel, Björn Nitzsche, Donald Lobsien, Johannes Seeger, Holm Schneider, Johannes Boltze

**Affiliations:** 1 Institute of Anatomy, Histology and Embryology, Faculty of Veterinary Medicine, University of Leipzig, Leipzig, Germany; 2 Division of Veterinary Anatomy, Vetsuisse Faculty, University of Bern, Bern, Switzerland; 3 Fraunhofer Institute of Cell Therapy and Immunology, Department of Cell Therapy, Leipzig, Germany; 4 Department of Neuroradiology, University Hospital of Leipzig, Leipzig, Germany; 5 Department of Pediatrics, University Hospital Erlangen, Erlangen, Germany; 6 Translational Centre for Regenerative Medicine, University of Leipzig, Leipzig, Germany; 7 Massachusetts General Hospital and Harvard Medical School, Neurovascular Regulation Laboratory at Neuroscience Center, Charlestown, Massachusetts, United States of America; Johns Hopkins University, United States of America

## Abstract

Cerebrovascular diseases are significant causes of death and disability in humans. Improvements in diagnostic and therapeutic approaches strongly rely on adequate gyrencephalic, large animal models being demanded for translational research. Ovine stroke models may represent a promising approach but are currently limited by insufficient knowledge regarding the venous system of the cerebral angioarchitecture. The present study was intended to provide a comprehensive anatomical analysis of the intracranial venous system in sheep as a reliable basis for the interpretation of experimental results in such ovine models. We used corrosion casts as well as contrast-enhanced magnetic resonance venography to scrutinize blood drainage from the brain. This combined approach yielded detailed and, to some extent, novel findings. In particular, we provide evidence for chordae Willisii and lateral venous lacunae, and report on connections between the dorsal and ventral sinuses in this species. For the first time, we also describe venous confluences in the deep cerebral venous system and an ‘anterior condylar confluent’ as seen in humans. This report provides a detailed reference for the interpretation of venous diagnostic imaging findings in sheep, including an assessment of structure detectability by *in vivo* (imaging) versus *ex vivo* (corrosion cast) visualization methods. Moreover, it features a comprehensive interspecies-comparison of the venous cerebral angioarchitecture in man, rodents, canines and sheep as a relevant large animal model species, and describes possible implications for translational cerebrovascular research.

## Introduction

Cerebrovascular diseases such as ischemic stroke and cerebral venous thrombosis (CVT) are major causes of mortality and neurological disabilities in adulthood. Thrombolysis is the most important and sometimes only therapeutic option for occlusive cerebrovascular diseases. While the treatment is restricted by a relatively narrow time window and a number of contraindications [1] in ischemic stroke, its use for cerebrovascular thrombosis remains a matter of debate [2], [3]. Thus, there is an unmet need for additional therapeutic options to arise from preclinical research. Predictive animal models are crucial to assess the safety and efficacy of novel therapeutic approaches for human patients [4]. During the last decade, large animal models of cerebrovascular diseases [5], [6] and neurosurgical interventions [7] became increasingly relevant. In particular, a number of sheep models emerged [8], [9] since this species was found highly practicable for translational research. Among such models, experimental middle cerebral artery occlusion has been described [10], [11] and, consequently the intracranial ovine arterial angioarchitecture has been studied in detail [12], [13]. However, little is known about the venous drainage in the sheep although important anatomical differences to other species including humans may limit the use of ovine cerebrovascular disease models. Since profound anatomical knowledge is an important prerequisite for translational research, the present study aimed to provide an in-depth analysis of the ovine intracranial venous blood system and its connections to extracranial veins. Beyond a detailed anatomical description including hitherto unknown structures in sheep and a comprehensive inter-species comparison of the venous vasculature, this study evaluates the applicability and accuracy of clinical imaging techniques to provide a reliable reference for further translational research on cerebrovascular pathologies in this species.

## Materials and Methods

### Ethics Statement and Vascular Corrosion Casting

All animal experiments (n = 14) were approved by the responsible federal animal welfare authority at the Regional Board Saxony, Detachment Leipzig, Department 24: veterinary affairs and animal welfare (protocol numbers TVV 33/04 and TVV 26/09) and performed in accordance with the guidelines of the European Convention for the Protection of Vertebrate Animals Used for Experimental and Other Scientific Purposes. Venous corrosion casts were prepared from Merino ewes (n = 10), previously subjected to experimental surgery unrelated to the head and the vascular system. Four additional animals were subjected to both MRI and CT imaging.

Sheep were sacrificed by an intravenous injection of 20% hydroxyl-butyramid, 5% mebezonium iodide, and 0.5% tetracaine hydrochloride (T61, Hoechst Roussel; 0.3 ml/kg body weight). After confirmed death, animals were decapitated between the second and third cervical vertebrae. The external jugular veins were carefully dissected and cannulated with 6-mm metallic bulb-headed cannulas followed by manual injection of methyl methacrylate (Kallocryl; Speiko – Dr. Speier GmbH, Germany). The heads were stored at 4°C for 24 hours to allow polymerisation of the injected resin. Maceration was performed with pepsin-hydrochloric acid and amylase/protease solution (Biozym SE, 10%) at 50°C until all soft tissues were dissolved. Thereafter, the skulls were trepanated and two paramedian bone plates of 9×4 cm were removed.

### Magnetic Resonance and Computed Tomography Vascular Imaging

Magnetic resonance venography (MRV) and computed tomography venography (CTV) were performed under general anaesthesia in four additional Merino ewes. Immediately after induction of anaesthesia, sheep were intubated and mechanically ventilated (Servo 900 D ventilator, Siemens, Germany). Table 1 provides details on medication schemes and contrast agents used.

**Table 1:** Medication schemes used throughout the study.

Purpose	Drug	Supplier	Application route	Dose/Concentration
Induction of anesthesia	2% xylazine hydrochloride (Xylazin)	Ceva Sante Animal	i.v. bolus	0.1 mg×kg^−1^
	ketamine hydrochloride (Ketamin)	Medistar	i.v. bolus	4 mg×kg^−1^
	midazolam (Midazolam)	Braun Melsungen	i.v. bolus	0.2 mg×kg^−1^
Inhalation anesthesia (during CTV)	Isoflurane	CP Pharma	ventilation	2.0%
	oxygen	Linde Medical Gases	ventilation	40%
Infusion anesthesia (during MRV)	Midazolam (Midazolam)	Braun Melsungen	i.v. infusion	0.1 mg×kg^−1^×h^−1^
	ketamine hydrochloride (Ketamin)	Medistar	i.v. infusion	2 mg×kg^−1^×h^−1^
	1%propofol (Propofol Lipuro)	Braun Melsunge	i.v. infusion	6 mg×kg^−1^×h^−1^
MRV contrast agent	gatoderic acid (Dotarem)	Guerbet, Cedex	i.v. bolus	20 ml per animal
CTV contrast agent	Iomeprol (Imeron350)	Bracco Imaging	i.v.	2 ml×s^−1^, 90 ml per animal, 80 s delay

Abbreviations: i.v.: intravenous, MRV: magnetic resonance venography, CTV: computed tomography venography.

Contrast enhanced MRV was performed using a 3T MRI Scanner (Magnetom Trio, Siemens, Erlangen, Germany) equipped with a surface flex coil. The following imaging parameters were applied: FLASH 3D, TR 3.25 s, TE 1.4 s, flip angle 19, slice thickness 1.2 mm, resulting in a voxel size of 0.5×0.5×1.2 mm.

CTV was performed with a 64 channel MDCT (Brilliance 64, Philips Healthcare, Hamburg, Germany) under the following imaging parameters: 0.8 mm slice thickness, 0.4 mm increment, 120 kV, 300 mAs, resulting in a voxel size of 0.5×0.5×0.8 mm. Imaging reconstructions and further processing were conducted using the Osirix 2.8 freeware (Osirix, Geneva, Switzerland).

### Comparison of Structure Detectability

To investigate sensitivity and specificity of corrosion casts and MRV, a semi-quantitative score system was applied. The visualization of venous structures was categorized as not detectable (−), barely visible (+), moderately visible (++) and distinctly visible (+++). In cases not allowing clear interpretation, CTV was performed as an independent imaging modality.

## Results

All structure designations and abbreviations are given in table S1, providing termini as used in the Nomina Anatomica Veterinaria (NAV) and in both the Nomina Anatomica (NA) and the Terminologia Anatomica (TA).

### Morphology of the Dorsal Sagittal Sinus

**Figure 1:**
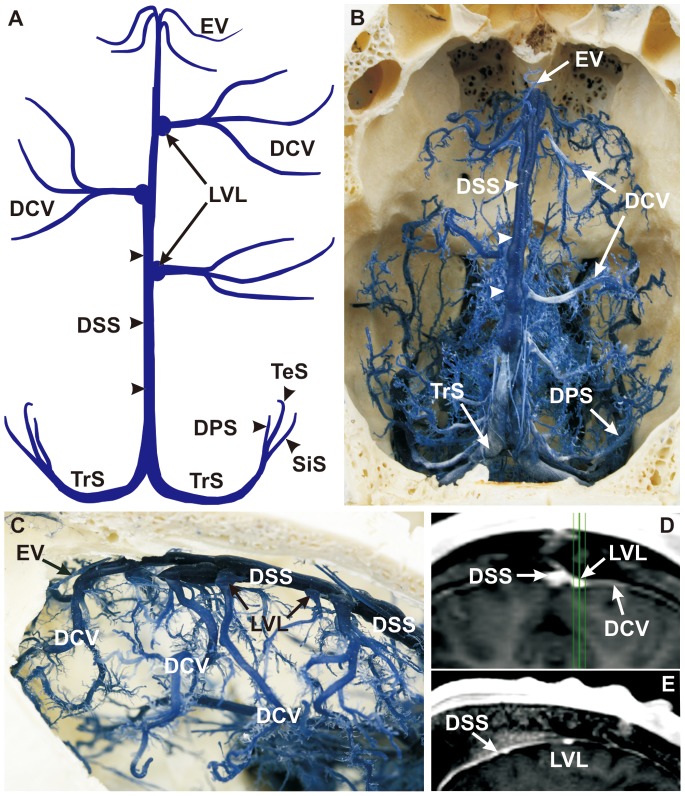
Morphology of the dorsal sagittal sinus. (**A**) Schematic drawing of corrosion cast, dorsal view. (**B**) Corrosion cast, dorsal view. The dorsal sagittal sinus (DSS) was the major cerebral venous drainage of the dorsal sinus system. Along its course, the DSS received ethmoidal, cerebral, meningeal, and diploic veins from the skull. Its superficial profile showed an axial groove over the whole length and nodular protrusions on the caudal one-third of the DSS (B, arrowheads). (**C**) Corrosion cast, lateral left view. The confluences of the dorsal cerebral veins (DCV) with the DSS showed pinhead-like openings called lateral venous lacunae (LVLs; A, C, D, E) and providing cerebrospinal fluid drainage into the venous system. (**D**) MRV, coronal section (maximum intensity projection of E, green). (**E**) MRV, sagittal section. The LVLs are interlinked between the DCV and the DSS in two different sections of MRV. **DCV:** dorsal cerebral vein, **DPS:** dorsal petrosal sinus, **DSS:** dorsal sagittal sinus, **EV:** ethmoidal vein, **LVLs:** lateral venous lacunae, **SiS:** sigmoid sinus, **TeS:** temporal sinus, **TrS:** transverse sinus.

**Figure 2:**
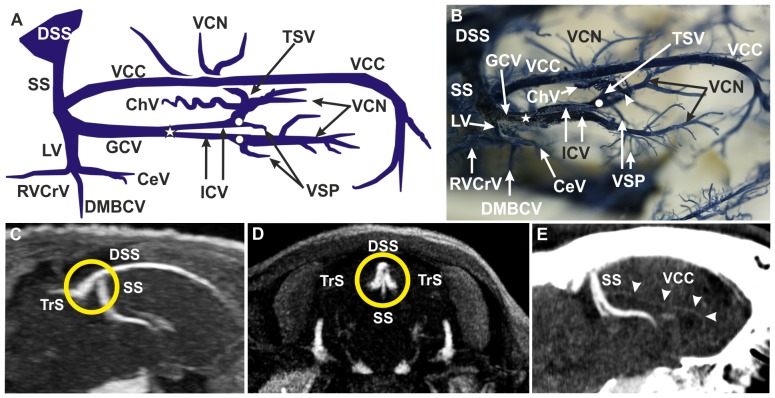
Deep cerebral venous system. (**A**) Schematic drawing of corrosion cast, lateral right view. The straight sinus (SS) was the venous drainage route of the medial cortex, the corpus callosum, the basal ganglia and part of the diencephalon (A–E). (**B**) Corrosion cast, dorsolateral right view. The unpaired great cerebral vein (GCV) was formed by both internal cerebral veins (ICV) in the ‘confluens venosus caudalis’ (A, B; white star). The vein of corpus callosum (VCC) and the lateral vein (LV), with three tributaries, drained into the GCV. The ICVs originated from two distinct converging branches in the ‘confluens venosus rostralis’ (A, B; white dots): the vein of the septum pellucidum (VSP) and the thalamostriate vein (TSV). The TSVs received inflow from caudate nucleus veins (VCN; A, B) and from the choroidal vein (ChV; A, B). (**C**) MRV, sagittal section. (**D**) MRV, coronal section. The confluence of sinuses (orange circle; C, D) referred to the crossroad between the DSS, the SS and both transverse sinuses (TrS). (**E**) CTV, sagittal section. The VCC was large and conspicuous in the corrosion cast (A, B), but much less prominent in CTV imaging (E, arrowheads). **CeV:** central vein, **ChV:** choroidal vein, **DMBCV:** dorsomedial basilar cerebral vein, **DSS:** dorsal sagittal sinus, **GCV:** great cerebral vein, **ICV:** internal cerebral vein, **LV:** lateral vein, **RVCrV:** rostral ventral cerebellar vein, **SS:** straight sinus, **TrS:** transverse sinus, **TSV:** thalamostriate vein, **VCC:** vein of the corpus callosum, **VCN:** vein of caudate nucleus, **VSP:** vein of the septum pellucidum. Please note: right TSV removed for better insight and interpretation in A and B.

The dorsal sagittal sinus (DSS, Fig. 1A, 1B) collects blood from the dorsal parts of the brain and skull. It originates at the crista galli of the ethmoid bone (Fig. 1B) and runs within the falx cerebri to the internal occipital protuberance (Fig. 1B, 1C; for MRV see Fig. 7B). Corrosion casts unveiled a longitudinal median groove over its entire course. Nodular protrusions were seen in the caudal third, but not in the rostral part (Fig. 1B). Corrosion casts also provided evidence of trabecular structures (chordae Willisii) within the DSS, which prevented a complete filling of the vascular lumen. Due to those structures, discrete foci of contrast agent filling defects were detected in axial MRV projections (Fig. 6B). The DSS received inflow from the ethmoidal veins (EV) (rostrally; Fig. 1A –1C; for MRV see Fig. 5B and Fig. 6D), the dorsal cerebral veins (DCV) (laterally; Fig. 1A –1D; for MRV see Fig. 5C and Fig. 6B) and both diploic and meningeal veins (dorsally). DCV were connected to lateral, pinhead-size expansions of lateral venous lacunae (LVLs) of the DSS (Fig. 1A, 1C –1E). The DSS received the straight sinus (SS) and split into the bilateral transverse sinus (TrS) (Fig. 1A, 1B). This point of venous confluence is called confluence of sinus (Fig. 2C, 2D).

### Deep Cerebral Venous System

Both thalamostriate veins (TSVs) were identified by their convergent, dorsally convex course in a rostromedial direction in corrosion casts and MRV images. It was clearly visible in corrosion casts that each TSV received further venous input from the vein of the septum pellucidum (VSP), the choroidal vein (ChV) and the veins of caudate nucleus (VCN) (Fig. 2A, 2B). The VSP, running rostroventrally to caudodorsally into the TSV, and the ChV being characterized by its typical ‘brush-like’ appearance (numerous short branches) (Fig. 2A, 2B), were clearly identified in corrosion casts but not by MRV. Likewise, the VCNs were only visible in corrosion casts where they were found to drain draining into the TSVs from rostral and lateral directions (Fig. 2A, 2B). The coalescence of the TSV and the VSP on each side formed the paired internal cerebral veins (ICV) (Fig. 2A, 2B) at the ‘confluens venosus rostralis’. TSVs and their continuation into the ICV were particularly apparent in MRV images (Fig. 7B –7D). After bending backwards, the ICVs merged into the unpaired great cerebral vein (GCV) at the ‘confluens venosus caudalis’. Both veins were clearly visible in corrosion casts as well as in MRV. The GCV was found to be about 1.5 mm in diameter and between 7.0 and 8.0 mm in length. It runs caudally between the junctions of the ICVs and the vein of corpus callosum (VCC) (Fig. 2A, 2B). The GCV received input from the paired lateral veins (LV) of the lateral ventricle and the unpaired VCC. The VCC was observed as a large and conspicuous vein of about 4 cm length in corrosion casts (Fig. 2B). It appeared as a very small vessel in CTV (Fig. 2E) and was undetectable in MRV (Fig. 7B). Each LV received three tributaries: a central vein (CeV) taking its course rostrally, a dorsomedial basilar cerebral vein (DMBCV) running ventrally and a rostral ventral cerebellar vein (RVCrV) taking its course caudally (Fig. 2A, 2B). The DMBCV approached the ventral cerebral veins, but no anastomoses between these vessels were observed in our study. The fine LV branches could only be discriminated in corrosion casts, but not by *in vivo* imaging. After entering the falx cerebri, the GCV continued as the SS which ventrally joined the caudal third of the DSS (Fig. 2A –2D; for MRV see Fig. 5F, 5G; Fig. 7B).

### Ventral Cerebral Veins

**Figure 3:**
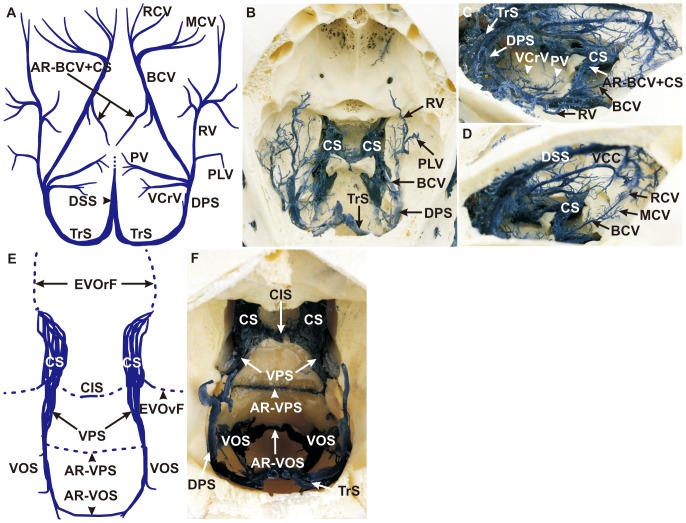
Ventral cerebral veins and the anastomoses of the ventral sinus system. (**A**) Schematic drawing of corrosion cast, dorsal view. The ventral cerebral veins included the basilar cerebral vein (BCV) and the rhinal vein (RV), which joined the dorsal petrosal sinus (DPS) as the main drainage system of the ventral cerebral veins,. (**B**) Corrosion cast, dorsocaudal view. The piriform lobe vein (PLV) drained into the RV (A, B) whereas the pontine vein (PV) and the ventral cerebellar vein (VCrV) joined the DPS (A, C) (**C**) Corrosion cast, dorsolateral right view. An anastomotic ramus projected from the BCV into the cavernous sinus (CS; AR−BCV+CS; A, C). (**D**) Corrosion cast, right caudodorsolateral view. The rostral cerebral veins (RCV) and the middle cerebral veins (MCV) drained into the BCV. (**E**) Schematic drawing of corrosion cast, dorsal view. The ventral sinus system includes three main bilaterally symmetrical sinuses, whereas all sinuses had an interconnection with their opposite side. (**F**) Corrosion cast, dorsorostral view. From rostral to caudal: the CS with the caudal intercavernous sinus (CIS), the ventral petrosal sinus (VPS) with an intraosseus anastomotic ramus of the VPS (AR-VPS) and the ventral occipital sinus (VOS) with an anastomotic vein (AR-VOS), which pass the foramen magnum ventrally. **AR−BCV+CS:** anastomotic ramus of the BCV and CS, **AR−VOS**: anastomotic ramus of the VOS, **AR−VPS**: anastomotic ramus of the VPS, **BCV:** basilar cerebral vein, **CIS**: caudal intercavernous sinus, **CS:** cavernous sinus, **DPS:** dorsal petrosal sinus, **DSS:** dorsal sagittal sinus, **EVOrF:** emissary vein of the foramen orbitorotundum, **EVOvF:** emissary vein of oval foramen, **MCV:** middle cerebral vein, **PLV:** piriform lobe vein, **PV**: pontine vein, **RCV:** rostral cerebral vein, **RV:** rhinal vein, **TrS:** transverse sinus, **VCC:** vein of corpus callosum, **VCrV**: ventral cerebellar vein, **VOS**: ventral occipital sinus, **VPS:** ventral petrosal sinus.

The basilar cerebral vein (BCV), being situated at the ventral brain base, received inflow from the rostral cerebral vein (RCV) and from the middle cerebral vein (MCV). This situation was clearly visible in corrosion casts (Fig. 3A, 3D), but not in MRV images. In the further course, the rhinal vein (RV) and the piriform lobe vein (PLV) formed a main outflow track which drained to the dorsal petrosal sinus (DPS) together with the BCV (Fig. 3A, 3B). The RCV arose from fine branches in the rostral cranial fossa near the ventral part of the crista galli (Fig. 3A, 3D), run caudally over the orbitosphenoidal crest and merged with the MCV (Fig. 3A, 3D), forming the BCV. The latter started dorsally of the optic canal (Fig. 3A, 3C, 3D). Some minor vessels took their course from the BCV in a ventromedial direction and anastomosed with the cavernous sinus (CS) (AR−BCV+CS) (Fig. 3A, 3C, for MRV see Fig. 5G) as detected in corrosion casts. The RV proceeded along the cerebral juga on the temporal brain surface (Fig. 3A–3C) and ran in a caudally convex arch, receiving input from the PLV before joining the DPS together with the BCV (Fig. 3A–3C). Despite being clearly detectable in corrosion casts, both veins were barely identifiable on MRV images (Fig. 5D, 5F; Fig. 6F, 6G; Fig. 7C–7G). The pontine vein (PV) and the ventral cerebellar veins (VCrV) also joined the DPS. The former emerged at the pontine impression and ran in a caudolateral direction to reach the DPS (Fig. 3A, 3C) rostrally of the confluence of the VCrV (Fig. 3A, 3C). The PV and the VCrV could not be visualized in MRV. The paired DPS joined the ipsilateral transverse sinus and were well detectable in corrosion casts (Fig. 3A –3C), and, to a limited extent, in MRV (Fig. 5H; Fig. 6D–6G; Fig. 7D–7F).

### Anastomoses of the Ventral Sinus System

The ventral sinus system encompassed a bilateral system of three main sinuses in the middle and caudal cranial fossae (from rostral to caudal): (1) the cavernous sinus (CS), (2) the ventral petrosal sinus (VPS) and (3) the ventral occipital sinus (VOS) (Fig. 3E, 3F; for MRV see Fig. 5E–5H, 5K; Fig. 6F–6J; Fig. 7B–7E). The paired CS displayed a plexiform structure which extended from the foramen orbitorotundum to the dorsum sellae. The caudal intercavernous sinus (CIS), a prominent anastomosis between caudal parts of the CS, was clearly identifiable in corrosion casts (Fig. 3E, 3F) and MRV (Fig. 5F; Fig. 6J). A rostral intercavernous sinus could not be detected in any specimen. The CS emptied via an emissary vein through the foramen orbitorotundum (EVOrF), connecting the CS to the extracranial ophthalmic plexus (OP) (Fig. 6I). Another anastomotic branch between CS and extracranial pterygoid plexus (PP) (AR−CS+PP), which was also found to run through the foramen orbitorotundum before turning laterally to the PP, was observed in corrosion casts (data not shown) as well as in MRV (Fig. 5D; Fig. 6J; Fig. 7D). This anastomosis has not been reported previously. The emissary vein of the oval foramen (EVOvF) was connected to the PP as well (Fig. 3E; for MRV see Fig. 5F, Fig. 6J; Fig. 7C). The CS continued caudally as the paired VPS which proceeded in a caudo-ventral direction. Before reaching the jugular foramen, the sinus was found to connect with its fellow, the anastomotic ramus of the VPS (AR-VPS) (Fig. 3E, 3F). The small-calibre AR-VPS was situated within the occipital bone between the pontine and medullary impressions and only visible in corrosion casts after bone removal. The VPS tapered and partly emptied into the emissary vein of the jugular foramen. The paired VOS, representing the caudal extension of the VPS, were connected by an anastomotic vein which passed the ventral boundary of the foramen magnum, the anastomotic ramus of the VOS (AR-VOS) (Fig. 3E, 3F; for MRV see Fig. 5K; Fig. 6I).

### Course of the Emissary Veins of the Temporal Sinus and Formation of the Anterior Condylar Confluent

**Figure 4:**
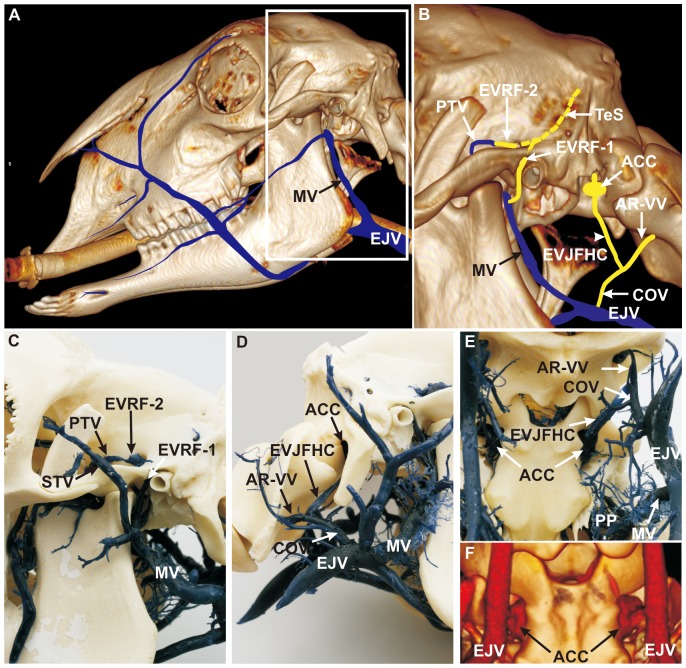
Course of the emissary veins of the temporal sinus and the formation of the anterior condylar confluent. (**A**) 3D CT reconstruction of the head combined with schematic vein drawings (blue), lateral, left view. White frame: inset of B. The ovine extracranial veins can be observed in this view, particularly the outer drainage system of the intracranial veins. (**B**) 3D CT scan combined with schematic diagram of blue and orange colored veins (interrupted orange vein of temporal sinus shows the invisible part of the sinus in the temporal meatus), lateral left view (paracondylar process removed). (**C**) Corrosion cast, lateral left view. The temporal sinus (TeS) ran through the temporal meatus and split into two distinct vessels, (1) the first emissary vein (EVRF-1), which left the main opening of the retroarticular foramen and joined the maxillary vein (MV), and (2) the second emissary vein (EVRF-2) which ran next to a tributary canal, passed a tributary foramen, and joined the profundal temporal vein (PTV). (**D**) Corrosion cast, lateral right view. (**E**) Corrosion cast, ventrolateral view. The emissary vein of the jugular foramen and the emissary vein of the hypoglossal canal converged towards an extracranial orifice and formed the ‘anterior condylar confluent’ (ACC). The emissary vein of the jugular foramen and hypoglossal canal (EVJFHC) merged with an anastomotic ramus of the vertebral vein (AR-VV), to form the craniooccipital vein (COV), which drained into the external jugular vein (EJV). (**F**) 3D CT scan, ventrolateral view. The ACC is a clearly visible structure in the sheep. **ACC**: anterior condylar confluent, **AR-VV**: anastomotic ramus of the vertebral vein, **COV**: craniooccipital vein, **EJV**: external jugular vein, **EVJFHC**: emissary vein of the jugular foramen and hypoglossal canal, **EVRF-1:** first emissary vein of retroarticular foramen, **EVRF-2:** second emissary vein of retroarticular foramen, **MV**: maxillary vein, **PP:** pterygoid plexus, **PTV**: profundal temporal vein, **STV:** superficial temporal vein, **TeS**: temporal sinus.

**Figure 5:**
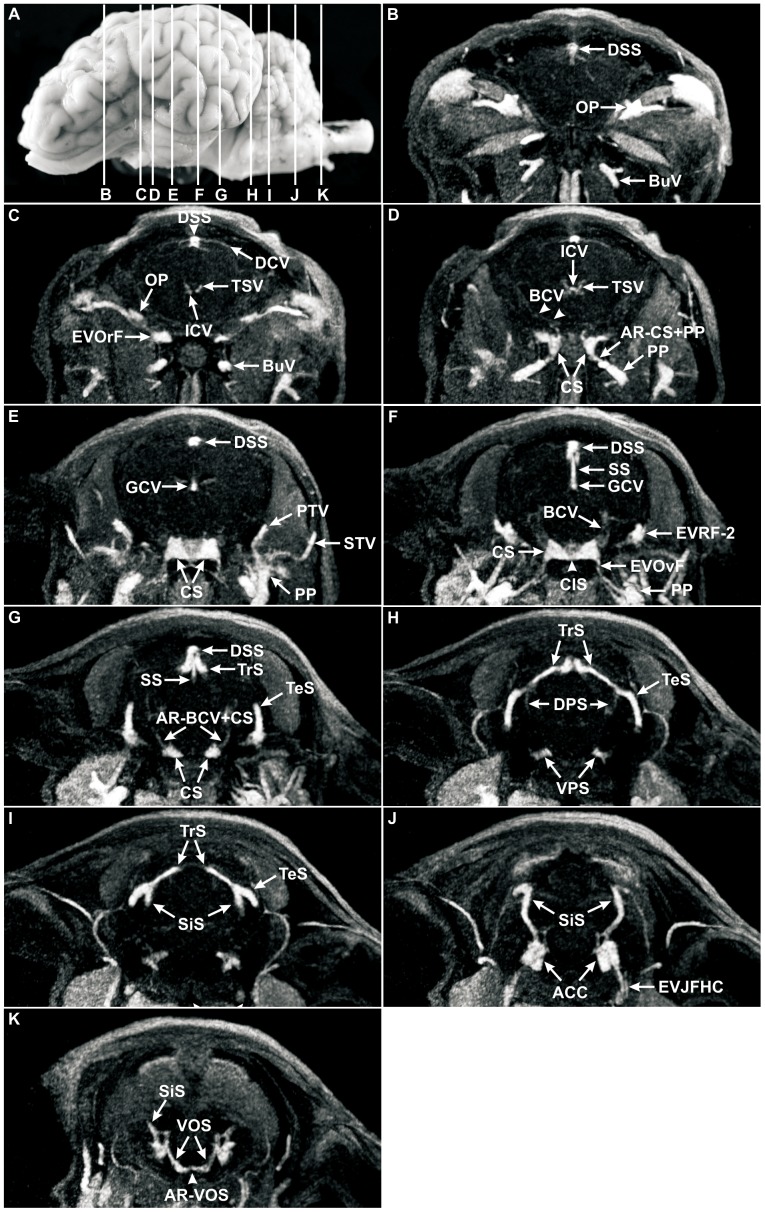
Coronal MRV slices through the head of a sheep after intravenous injection of Gadolinium (Dotarem). (**A**) Lateral, left view of the ovine brain. Lines delineate the levels of coronal MRV slices, letters correspond to the letters in coronal MRV images; **B–K,** MRV, coronal sections. The angioarchitecture of the dorsal and ventral sinus systems including their connecting veins was clearly depicted after contrast injection of Gadolinium (Dotarem) in comparison to the deep and ventral cerebral veins. Note the completely visible signals of the dorsal sagittal sinus (DSS; B–G), the transverse sinus (TrS; G–I), the temporal sinus (TeS; G–I), the sigmoid sinus (SiS; I–K), the cavernous sinus (CS; D–G), the ventral petrosal sinus (VPS; H), the ventral occipital sinus (VOS; K), the anterior condylar confluent (ACC; J), as well as the great cerebral vein (GCV; E, F) and the straight sinus (SS, F, G). Note the faint signal of the thalamostriate veins (TSV; C, D), the internal cerebral veins (ICV, D), the basilar cerebral vein (BCV), and the dorsal petrosal sinus (DPS; D, F, H). Two anastomotic rami could be seen in MRV strongly: the anastomotic ramus between cavernous sinus and pterygoid plexus (AR-CS+PP; D) with a highly signal intensity and the anastomotic ramus between BCV and CS (AR-BCV+CS; G) as a diminutive vessel. **ACC:** anterior condylar confluent, **AR−BCV+CS:** anastomotic ramus between basilar cerebral vein and cavernous sinus, **AR−CS+PP**: anastomotic ramus between cavernous sinus and pterygoid plexus, **AR−VOS:** anastomotic ramus between both ventral occipital sinus, **BCV**: basilar cerebral vein, **BuV:** buccal vein, **CIS:** caudal intercavernous sinus, **DCV:** dorsal cerebral vein, **DPS:** dorsal petrosal sinus, **DSS**: dorsal sagittal sinus, **EVJFHC:** emissary vein of jugular foramen and hypoglossal canal, **EVOrF:** emissary vein of the foramen orbitorotundum, **EVOvF:** emissary vein of oval foramen, **EVRF-2:** second emissary vein of retroarticular foramen, **GCV:** great cerebral vein, **ICV:** internal cerebral vein, **OP:** ophthalmic plexus, **PTV:** profundal temporal vein, **RV**: rhinal vein, **SiS**: sigmoid sinus, **SS:** straight sinus, **STV:** superficial temporal vein, **TeS:** temporal sinus, **TrS:** transverse sinus, **TSV:** thalamostriate vein, **VOS:** ventral occipital sinus, **VPS:** ventral petrosal sinus.

**Figure 6:**
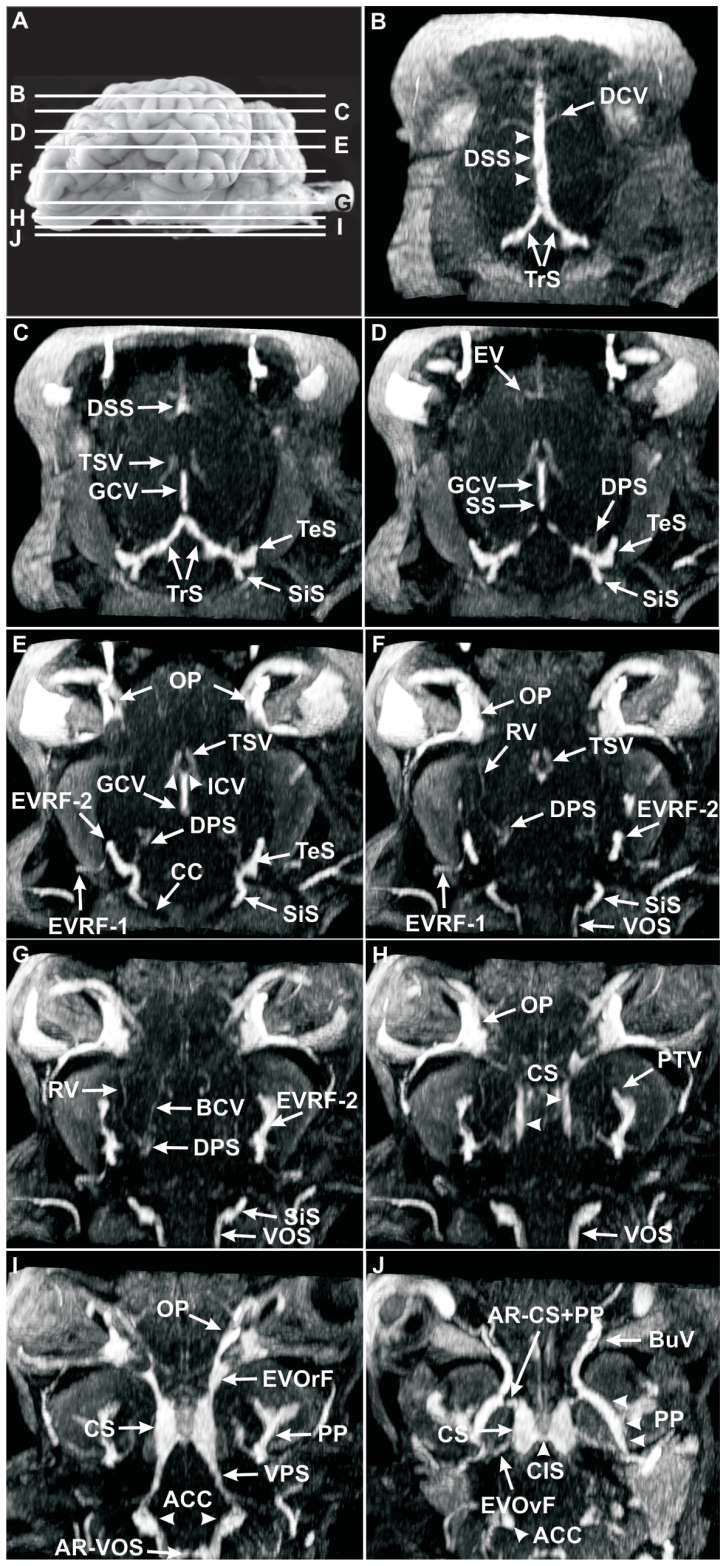
Axial MRV slices through the head of a sheep after intravenous injection of Gadolinium (Dotarem). (**A**) Lateral left view of the ovine brain. Lines delineate the levels of axial MRV slices, letters correspond to the letters in the axial MRV images; **B–J**, MRV, axial sections. Note the irregular filling of the completely visible dorsal sagittal sinus (DSS) after intravenous injection of Gadolinium (Dotarem) as a result of the chordae Willisii (B). A better detectability of the deep and ventral cerebral veins can be observed in the axial slices. The great cerebral vein (GCV) and the straight sinus (SS) were completely visible (C–E), the thalamostriate vein (TSV) and the internal cerebral vein (ICV) were moderately visible (C–F), but no evidence of the vein of septum pellucidum (VSP) or choroidal veins (ChVs) was found. The visualization of the basilar cerebral vein (BCV) and the rhinal vein (RV) were barely visible and the dorsal petrosal sinus (DPS) was moderately conspicuous in MRV (C–G). The first emissary vein of retroarticular foramen (EVRF-1) was a moderately visible vein (E, F). The second emissary vein of retroarticular foramen (EVRF-2) was a noticeable vessel which passed the tributary canal of the temporal meatus and drained into the profundal temporal vein (PTV; E–H). The butterfly-shaped cavernous sinus (CS) could be regarded as a venous crossroad with star-shaped connections to intra- and extracranial veins (I, J). From rostral to caudal, these were: (1) the emissary vein of orbitorotund foramen (EVOrF; I) with the ophthalmic plexus (OP; I); (2) the anastomotic ramus between cavernous sinus and pterygoid plexus (PP) (AR-CS+PP; J); (3) the emissary vein of the oval foramen (EVOvF; J) with the PP; (4) the ventral petrosal sinus (VPS; I) and (5) the caudal intercavernous sinus (CIS; J). Note the clear delineation of the anterior condylar confluent (ACC; I). **ACC:** anterior condylar confluent, **AR−CS+PP**: anastomotic ramus between cavernous sinus and pterygoid plexus, **AR−VOS:** anastomotic ramus between both ventral occipital sinus, **BCV**: basilar cerebral vein, **BuV:** buccal vein, **CC:** condylar canal, **ChV:** choroidal veins, **CIS:** caudal intercavernous sinus, **CS:** cavernous sinus, **DCV:** dorsal cerebral vein, **DPS:** dorsal petrosal sinus, **DSS**: dorsal sagittal sinus, **EV:** ethmoidal vein, **EVOrF**: emissary vein of the foramen orbitorotundum, **EVOvF:** emissary vein of oval foramen, **EVRF-1:** first emissary vein of retroarticular foramen, **EVRF-2:** second emissary vein of retroarticular foramen, **GCV**: great cerebral vein, **ICV:** internal cerebral vein, **OP**: ophthalmic plexus, **PP**: pterygoid plexus, **RV**: rhinal vein, **SiS:** sigmoid sinus, **SS**: straight sinus, **TeS:** temporal sinus, **TrS:** transverse sinus, **TSV:** thalamostriate vein, **VOS:** ventral occipital sinus, **VPS:** ventral petrosal sinus **VSP**: vein of septum pellucidum.

**Figure 7:**
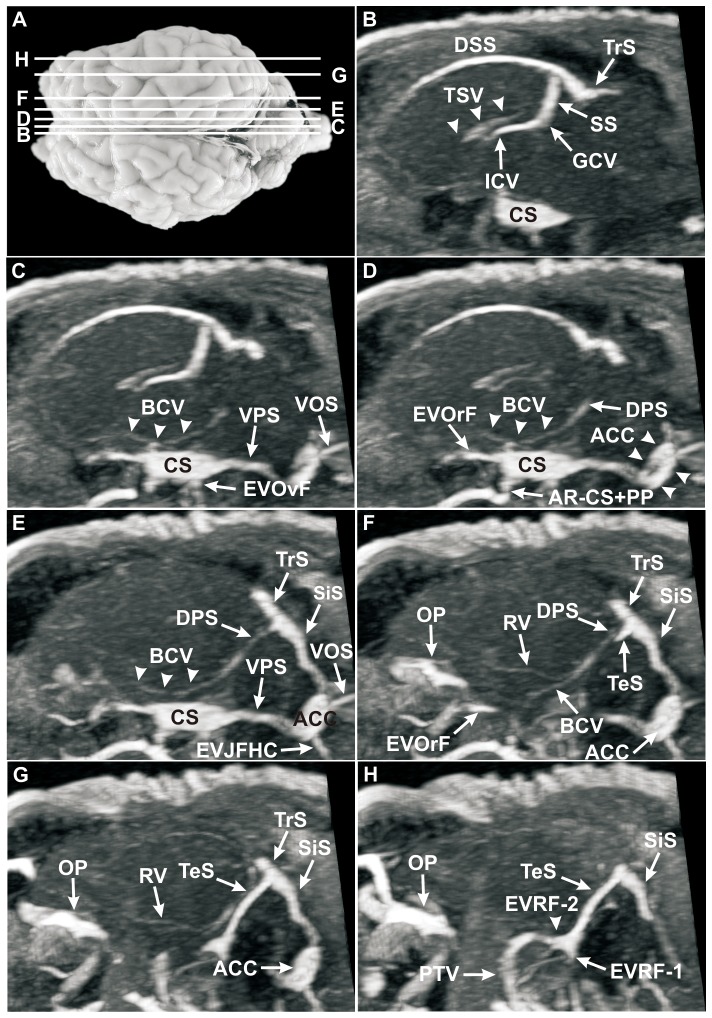
Sagittal MRV slices through the head of a sheep after intravenous injection of Gadolinium (Dotarem). (**A**) Dorsal view of the ovine brain. Lines delineate the levels of sagittal MRV slices, letters correspond to the letters in sagittal MRV images; **B–H**, MRV, sagittal sections. The sagittal sections provided a detailed depiction of all intracranial sinuses and veins, also the deep and ventral cerebral veins and particularly the confluence of sinus (B). Interestingly, the vein of corpus callosum (VCC) could not be detected in MRV. The thalamostriate vein (TSV; B, arrowheads) showed a cornu-like course and drained into the conspicuous internal cerebral vein (ICV; B). The weakly filled basilar cerebral vein (BCV; C, D, E, arrowheads) could easily be identified over a longer distances. BCVs and rhinal veins (RVs) drained through the dorsal petrosal sinus (DPS; D, E, F) into the transverse sinus (TrS; E, F, G). The TrS split into the temporal sinus (TeS) and the sigmoid sinus (SiS; F–H). Note the clear illustration of the TrS, TeS, SiS and the anterior condylar confluent (ACC; D–G). The ACC offered a large extent in these sagittal sections (D, arrowheads). The first (EVRF-1) and the second (EVRF-2; H, arrowhead) emissary vein of retroarticular foramen are clearly visible. **ACC:** anterior condylar confluent, **AR−CS+PP:** anastomotic ramus between cavernous sinus and pterygoid plexus, **BCV**: basilar cerebral vein, **CS:** cavernous sinus, **DPS:** dorsal petrosal sinus, **DSS:** dorsal sagittal sinus, **EVJFHC:** emissary vein of jugular foramen and hypoglossal canal, **EVOrF:** emissary vein of foramen orbitorotundum, **EVOvF:** emissary vein of oval foramen, **EVRF-1:** first emissary vein of retroarticular foramen, **EVRF-2:** second emissary vein of retroarticular foramen, **GCV:** great cerebral vein, **ICV:** internal cerebral vein, **OP:** ophthalmic plexus, **PTV:** profundal temporal vein, **RV**: rhinal vein, **SiS:** sigmoid sinus, **SS:** straight sinus, **TeS:** temporal sinus, **TrS:** transverse sinus, **TSV:** thalamostriate vein, **VCC:** vein of the corpus callosum, **VOS:** ventral occipital sinus, **VPS:** ventral petrosal sinus.

On either side, the transverse sinus (TrS) split into a rostral temporal sinus (TeS) and a caudal sigmoid sinus (SiS), clearly visible in corrosion casts (Fig. 1A, 1B) and on MRV images (Fig. 5G –5J; Fig. 6C; Fig. 7E –7H). The TeS entered the temporal meatus in a rostro-ventral direction where it split into two distinct vessels which emerged as emissary veins from the retroarticular foramen. The first emissary vein ran through the main retroarticular foramen (EVRF-1) and joined the maxillary vein (MV) near the confluence of the superficial temporal vein (STV) (Fig. 4B, 4C; for MRV see Fig. 6E, 6F; Fig. 7H). The prominent second emissary vein of the tributary retroarticular foramen (EVRF-2) was found along the tributary canal inside the temporal fossa and joined the deep temporal vein (PTV) as seen in both corrosion casts (Fig. 4B, 4C) and MRV (Fig. 5F; Fig. 6E, Fig. 7H). The SiS passed the condylar canal (CC) and drained into the VOS (Fig. 6E). Thus, the SiS connected the dorsal with the ventral sinus system. The VOS emptied into the internal vertebral venous plexus and into the emissary vein of the hypoglossal canal which left the skull through the hypoglossal canal. At the extracranial opening, the emissary veins of the jugular foramen and of the hypoglossal canal merged into a conspicuous, plexus-like structure which was detectable in corrosion casts (Fig. 4B, 4D, 4E) and on MRV images (Fig. 5J; Fig. 6I, 6J; Fig. 7D–7G). This structure is described as the anterior condylar confluent (ACC) in humans. It was located between the occipital condyle and the paracondylar process of the occipital bone. The ACC was concealed by the paracondylar process (Fig. 4D) in the lateral view. The ACC emptied into the emissary vein of the jugular foramen and hypoglossal canal (EVJFHC). The latter received the anastomotic ramus of the vertebral vein (AR-VV) prior to continuing as the craniooccipital vein (COV) which finally connected to the external jugular vein (EJV, Fig. 4B, 4D –4E).

### Detectability of Cerebral Venous Structures by the Techniques Applied

A synopsis of the visibility of individual vessels in corrosion casts and by means of MRV is summarized in table 2. In principle, corrosion casts provide a superior visibility, particularly of very small structures. CTV could, to some extent, be used to compensate for occasional lack of detectability on MRV imaging.

**Table 2:** Comparison of structure detectability by corrosion casting and MRV.

Venous structure	Abbreviation	CC	MRV	Comments
anastomotic ramus of basilar cerebral vein+cavernous sinus	AR-BCV+CS	++	+	
anastomotic ramus of cavernous sinus+pterygoid plexus	AR-CS+PP	−*	+++	*not visible in corrosion casts (situated within skull bone)
anterior condylar confluent	ACC	+++	+++	
basilar cerebral vein	BCV	+++	+	
caudal intercavernous sinus	CIS	+++	++	
cavernous sinus	CS	+++	+++	no sign of rostral inter-cavernous sinus
central vein	CeV	++	−	
choroidal vein	ChV	+++	−	
dorsal cerebral vein	DCV	+++	+/++	
dorsal petrosal sinus	DPS	+++	+/++	
dorsal sagittal sinus	DSS	+++	+++	resin profiles in corrosion cast and irregular fillings in MRV– chordae Willisii
dorsomedial basilar cerebral vein	DMBCV	++	−	
emissary vein of retroarticular foramen (1. branch)	EVRF-1	+++	++	
emissary vein of retroarticular foramen (2. branch)	EVRF-2	+++	+++	
emissary vein of foramen orbitorotundum	EVOrF	+++*	+++	*outside of the foramen orbitorotundum
emissary vein of oval foramen	EVOvF	++*	++	*outside of oval foramen
ethmoidal vein	EV	+++	−	
great cerebral vein	GCV	+++	+++	
internal cerebral vein	ICV	+++	++/+++	
lateral vein	LV	+++	−	
lateral venous lacunae	LVL	+++	+++	liquor drainage
middle cerebral vein	MCV	+++	−	
piriform lobe vein	PLV	+++	−	
pontine vein	PV	++	−	
profundal temporal vein	PTV	+++	+++	
rhinal vein	RV	+++	+	
rostral cerebral vein	RCV	+++	−	
rostral ventral cerebellar vein	RVCrV	++	−	
sigmoid sinus	SiS	+++*	+++	*outside of condylar canal
straight sinus	SS	+++	+++	
temporal sinus	TeS	+++*	+++	*outside of temporal meatus
thalamostriate vein	TSV	+++	++	
transverse sinus	TrS	+++	+++	
vein of corpus callosum	VCC	+++	−	only barely visible in CTV
vein of septum pellucidum	VSP	++	−	
veins of caudate nucleus	VCN	+++	−	
ventral cerebellar vein	VCrV	++	−	
ventral occipital sinus	VOS	+++	+++	
ventral petrosal sinus	VPS	+++	+++	

Overview on venous structures as seen in corrosion casts (CC) and MRV with respect to visibility of vessels (in alphabetical order). Visibility was rated by a score system: (−) not detectable; (+) barely visible; (++) moderately visible; (+++) distinctly visible. For structure denomination, please consult the table S1.

## Discussion

The major aim of the present study was to provide a comprehensive analysis of the ovine intracranial venous system by a combined approach using vascular corrosion casts and non-invasive *in vivo* imaging techniques. Relevant findings including hitherto not described anatomical structures are summarized in Fig. S1.

### Interspecies Comparison: Most Relevant Differences and Species Assessment

Table 3 provides a comprehensive overview on the cerebral venous angioarchitecture in sheep, humans, dogs, and rats. Significant differences in cerebral arterial blood supply exist between rodents and humans. For example, numerous inter-arterial anastomoses can prevent major cortical infarction after distal (cortical) middle cerebral artery (MCA) occlusion in most non-hypertensive rodent strains [4]. These anastomoses are almost completely missing in humans (and domestic mammals), and occlusion of the MCA usually has disastrous consequences. Hence, similar differences may be presumed for the venous system. The cerebral venous drainage in rats and mice as the predominant experimental species has not been described in much detail so far. Some information is available for the Sprague-Dawley rat [14], [15] which was used as a reference in our species comparison. Given the utmost importance of these species for basic cerebrovascular research and the lack of literature covering this topic, the rodent cerebral venous outflow tracks should be explored thoroughly in further studies.

**Table 3:** Interspecies comparison of venous structures in sheep, human, dogs and rats.

structure	sheep	human	dog	rat	comparison
**Morphology of the dorsal sagittal sinus**					
DCV	DCVs drainhemisphere surfaces,enter DSS [17], [27], [33], [40], [41]	Superior cerebral veins drain hemisphere surfaces, enter superior sagittal sinus [18]	DCV drain hemisphere surfaces,enter DSS [19], [20], [27], [42]	denominated as superficial cerebral veins, enter superiorsagittal sinus [15], [44]	sheep = human = dog = rat
DSS	DSS begins at crista galli, some fine EV drain intothe DSS, terminates into TrS [17], [27], [33], [40], [41]	denominated as superior sagittal sinus, anterior part sometimes absent, two superior cerebral veins replaced the sinus and functionally equivalent, sinus terminates into TrS [18], [25], [29]	DSS begins with large rostral dorsal cerebral veins, similar to sheep [19], [20], [27], [42]	denominated as superior sagittal sinus, similar tosheep [15], [43], [44]	sheep = human = dog = rat (except the origin)
LVLs	LVLs are lateral DCV expansions, connected to DSS, contain arachnoid granulations for cerebrospinal fluid drainage [41]	LVLs communicate with irregularly shaped venous spaces in the dura mater near the superior sagittal sinus (DSS equivalent) [22]	similar to sheep [20], [27]	n.d.	sheep = human = dog ≠ (?) rat
**Deep cerebral venous system**					
ChV	brush-like, with numerous short branches, drains TSV [27]	brush-like, with numerous shortbranches, drains TSV [45]	similar to sheep, but denominated as ‘thalamic vein’ [19], [20]	n.d.	sheep = human = dog ≠ (?) rat
GCV (Galen)	formed by ICV at ‘confluens venosus caudalis’ [17], [27]	formed by ICV and basal veins ofRosenthal forming at ‘confluensvenosus posterior’[18], [24], [45], [46]	formed by ICV [19], [20], [27], [42]	joins the aggregation of sinuses [43], [44]	sheep = human = dog = (?) rat
ICV	formed by TSV and VSP [17], [27]	formed by TSV and anteriorseptal veins [18], [24], [45]	similar to sheep [19], [20], [27]	similar to sheep [44]	sheep = human = dog = rat
LV	formed by CeV, DMBCV and RVCrV and drained intothe GCV	the lateral vein of lateral ventricle drain in theICV, is denominated as ‘lateral veinof lateral ventricle’ [49]	n.d.	n.d.	sheep ≠ human ≠ (?) dog ≠ (?)rat
SS	formed by confluence of GCV (ventrally), and VCC (dorsally); drains into confluence of sinus or TrS [17], [27], [41]	receives inferior sagittal sinus, the greatvein of Galen, and superior cerebellar veins;drains confluence of sinus or TrS[18], [24], [29]	similar to sheep [19], [20], [27], [42]	similar to sheep [15], [43], [44]	sheep = human = dog = rat
TSP	receives venous drainage from VCN, VSP, and ChV [17]	receives venous drainage from VCN(anterior and transverse),anterior septal vein, and ChV,denominated as ‘terminalvein’ [18], [45]	similar to sheep [19], [20]	n.d.	sheep = human = dog ≠ (?) rat
VCC	collects blood regions of ethmoid and rostral cranial fossa, and chiasmatic sulcus, drains into the SS [27], [41]	receives inflow from falx cerebri, upper surfaceof the corpus callosum, and medialsurfaces of the hemispheres,denominated as ‘inferior sagittalsinus’ [18], [29], [45]	similar to sheep [19], [20], [27]	similar to sheep, but denominated as ‘inferiorsagittal sinus’ [43], [44]	sheep = human = dog = rat
VCN	drains into TSV from rostrally and laterally	anterior and transverse caudate veins,drain into TSV [45]	n.d.	n.d.	sheep = human ≠ (?) dog ≠ (?) rat
VSP	connects with TSV, forming ICV on ‘confluens venosus rostralis’	denominated as ‘anterior septal vein’ or ‘septal vein’ [18], [45]	‘choroidal vein’ is functionallyand anatomically equivalent [20]	n.d.	sheep = human = dog ≠ (?) rat
**Ventral cerebral veins**					
BCV	formed by RCVs and MCVs, anastomosis to CS, enters DPS [17]	formed by anterior and deep middle cerebral, and inferior striate veins, terminates into ICV, SS or superior petrosal sinus, denominated as‘basal vein of Rosenthal’ [18], [24], [45], [46]	originates from several smallerveins, enters DPS	basal and rostral rhinal veins drain into the CS [15], [43], [44]	sheep = dog ≠ human ≠ rat
DPS	forms main outflow track from the ventral cerebral veins, joins the ipsilateral TrS[17], [27], [33], [41]	denominated as superior petrosal sinus,receives blood from the CS, from cerebellar and inferior cerebral veins [29]	similar to sheep [19], [27]	drains into the TrS [15], [44]	sheep = dog = (?) rat ≠ human
MCV	merges with RCV to form BCV, parallels MCA [17]	Deep middle cerebral veins form basal vein of Rosenthal with anterior cerebral and inferior striate veins [18], [46]	similar to sheep, denominated as‘deep middle cerebral vein’[20]	n.d.	sheep = human = dog ≠ (?) rat
superficial MCV	n.d.	starts at lateral hemispheric surface, ends in CS, sphenoparietal sinus or pterygoid plexus; anastomoses with veins of Trolard and Labbé [18], [24], [29]	n.d.	n.d.	only described in human
RCV	arises from fine branches in the rostral cranial fossa, projects caudally and merges with MCV, forming the BCV [17]	denominated as anterior cerebral veins, form basal vein of Rosenthal together with deep MCV [18], [24], [29]	similar to sheep [20]	anterior/superficial cerebral veins arise from ventral capillaries and drain into the superior sagittal sinus; there is a posterior superficial cerebral vein receiving blood from dorsal capillary system [15]	sheep = human = dog ≠ rat
RV	RV proceeds between impressions of the temporal and piriform lobes and receives input from the PLV before joining the DPS together with the BCV [17]	n.d.	not denominated anatomically,but potentially existing [20]	similar to sheep [43]	sheep = rat ≠ human ≠ dog
**Anastomoses of the ventral sinus system**					
BP	n.d.	Basilar venous plexus communicates with inferior petrosal, cavernous, and marginal sinus [48]	n.d.	n.d.	sheep ≠ human = rat ≠ dog
CS	paired CS, next to rostral epidural rete mirabile [17], [27], [33], [41]	paired CS, next to internal carotid artery [18], [45], [47]	paired CS, next to internalcarotid artery [19], [27], [42]	paired CS, next to internal carotid artery [14], [43], [44]	sheep = human = dog = rat
CIS	CS are transversally connected [17], [27], [41]	CS are transversally connected, complete ‘circular sinus’ [18], [47]	n.d. [19], [27], [42]	n.d.	sheep = human ≠ (?) dog ≠ (?) rat
RIS	n.d.	denominated as anterior intercavernous sinus, connects the two CS [18], [47]	inconstant [19, 27,42]	n.d.	sheep ≠ human ≠ dog ≠ (?) rat
VOS	caudal VPS continuation, receives input from SiS, drains into ACC and internal vertebral venous plexus. [17], [27]	n.d.; occipital sinus is functionally, but not anatomically equivalent [24]	similar to sheep[27]	n.d.	sheep = dog ≠ human ≠ (?) rat
VPS	caudal CS continuation, connects to ACC [17], [27], [33], [41]	receives inflow from CS, internal auditory vein, and veins from the medulla oblongata, pons, and cerebellum, denominated as ‘inferior petrosal sinus’ [18], [45]	similar to sheep[19], [27], [42]	n.d.	sheep = human = dog ≠ (?) rat
**Course of the emissary veins of the temporal sinus and formation of the anterior condylar confluent**					
ACC	formed by emissary vein of jugular foramen and emissary vein of hypoglossal canal, approximate size 10 to12 mm×7 mm, drains into the EVJFHC	formed by anterior and lateral condylar veins and branches of the internal jugular vein, the inferiorpetrosal sinus, the plexus of Rektorzik, and the prevertebral venous plexus, approximate size: 3 to 5 mm×2 mm. [38], [39]	n.d.	n.d.	sheep = human ≠ dog ≠ (?) rat (location); sheep ≠human ≠ dog ≠ (?) rat (inflow)
SiS	continued caudoventrally as caudal TrS branch, draining into VOS; connects dorsal and ventral sinus system. [17], [27], [33], [41]	continuation of the TrS at occipitopetrosal junction; drains into internal jugular vein [18], [24], [29]	similar to sheep[19], [27], [42]	similar to sheep (origin) and human (end), respectively [43]	sheep = human = dog = rat (origin); sheep ≠human = rat ≠ dog (end)
TeS	larger than SiS, merges with two emissary veins, drains into MV [27], [33], [41] and PTV	n.d.	similar to sheep[19], [27], [42]	functional equivalent: petrosquamosal sinus (but different anatomy)[14], [43], [44]	sheep = dog ≠ human≠ rat
TrS	DSS continuation, part ofconfluence of sinus [17, 27,33, 41]	caudal continuation of superior sagittal sinus, part of the confluence ofsinus [18], [24], [29]	similar to sheep[19], [27], [42]	similar to sheep [14], [15], [43], [44]	sheep = human = dog = rat
veins of Tro-lard, Labbé, Sylvius, and Rolando	n.d.	present [18], [24], [29]	n.d.	n.d.	only described in human

Structures are listed in alphabetical order. Abbreviations: n.d.: not described or absent,  = : similar/comparable to, ≠: not similar/not comparable to, (?): unsure (not described). The table is divided into subsections corresponding to paragraphs in the results and discussion.

Canines represent an important model species used in translational cerebrovascular and neurointerventional research, since the anatomy of the arterial blood supply to the brain is very similar to humans, though rich anastomoses may also exist in this species [16]. Importantly, dogs lack a rete mirabile, a plexiform arterial network arising from the maxillary artery and forming the internal carotid artery, which is common in artiodactyls. Canines are therefore a preferred species for experimental arterial intravascular procedures. Anatomical and physiological similarities of assessed venous structures in comparison to human anatomy were slightly lower in dogs (16 out of 30) as compared to sheep (19 out of 30). Almost half of the structures (13 out of 30) have not been described in the rat, indicating a significant lack of knowledge about the species. Hence, the present study indicates a close resemblance of the intracranial venous system between sheep and man. The following paragraphs discuss functional and anatomical differences in selected venous structures between large animals and humans in more detail.

### Morphology of the Dorsal Sagittal Sinus

The DSS is the main drainage system of the dorsal sinus system. In Merino sheep, it starts with the fusion of two to three dorsal rostral cerebral veins and collects blood from the DCV and from diploic veins [17]. This is similar to the situation in other domestic ruminants [17] and in humans, where the superior cerebral veins deliver blood to the superior sagittal sinus [18]. Variants have been described for dogs [19]. In canines, anastomoses between the rostral branches of the dorsal cerebral veins with branches of the RV have been reported [20], but no such variations were seen in our study. Moreover, some fine branches of ethmoidal veins delivered blood from the region of the crista galli to the DSS in our specimens. LVLs of the DSS have been described in humans [21], [22], monkeys [23], and dogs [20]. In humans, the LVLs collect blood predominantly from the meningeal veins, but not from the cortical veins [24]. In contrast, the LVL were found to connect the DCV with the DSS in Merino sheep. Several types of chordae Willisii have been distinguished by using standard anatomical methods [21], [25]. Our results corroborated these findings, with chordae displaying a trabecular, longitudinal or valve-like shape in sheep. Chordae Willisii in the median plane may functionally form a septum with trabecular and valve-like formations as seen in MRV. The latter are considered to prevent reverse blood flows and are also the most frequent type seen in humans [25]. In animals, the confluence of sinuses comprises the DSS, SS and TrS, whereas it encompasses the superior sagittal sinus, straight sinus, occipital sinus and transverse sinus in humans [26].

### Deep Cerebral Venous System

In humans, the SS is connected either with the confluence of sinuses or, more commonly, with the left TrS [24]. The SS connected with the confluence of sinuses in Merino sheep, which is congruent with previous findings [27]. The VCC was very prominent in ovine corrosion casts, but less prominent in MRV and CTV, and may easily be confused with the human inferior sagittal sinus. The LVs, previously undescribed for sheep, joined the great cerebral vein. This is in contrast to the human anatomy, were LVs drain into ICVs. Ovine LVs receive inflow from three tributaries, which directly drain into the GCV in canines [20], omitting LVs. The human equivalent of the ‘confluens venosus posterior’ is the ‘confluens venosus caudalis’. The ‘confluens venosus posterior’ in humans is formed of the GCV of Galen, as a merge of the internal cerebral veins and basal veins of Rosenthal [18]. In contrast, only the ICVs participate in the formation of the GCV in sheep. This formation has not been described in animals so far and, with respect to the human situation, may be denominated as ‘confluens venosus caudalis’. Another formation, called ‘confluens venosus anterior’ or ‘venous angle’ in humans, characterizes the confluence of the anterior septal vein with the TSV, giving rise to the internal cerebral vein [28], [29]. Of all subependymal veins, the TSV is best described since it is most evident in angiography [30]. The venous angle was formed by the point of origin of ICV at the thalamic tubercle, as seen in lateral views of cerebral angiograms by Kiliç & Akakin [29]. These authors also described anatomical variations regarding subependymal veins in the region of the foramen of Monro by means of MR time of flight venography. Here, MRV and CTV were proven ineffective to visualize the VSP, but Çimşit et al. [28] provided excellent images of the anterior septal vein by using MR time of flight which allows the detection of vessels with relatively slow blood flow.

### Ventral Cerebral Veins

The ventral cerebral veins are a group of veins which drain the rhinencephalon and enter the DPS, whereas the BCV is situated in the immediate vicinity of the arterial circle of Willis. This also applies to the RCV and MCV, which accompany the rostral and middle cerebral artery [17]. Several anastomoses to the more dorsal sinus system were noted in dogs [20]. In sheep, we found a correspondence to the RCV and the MCV. The basal vein in dogs continues on the lateral side of the cerebral peduncle and splits into dorsomedial and dorsolateral basal veins as described by Armstrong and Horowitz [20]. These authors also reported anastomoses between the dorsomedial basal vein and the GCV as well as between the basal vein and the cavernous sinus.

The first anastomosis could not be detected in our specimens. However, we found an anastomosis between the BCV and the CS. The basal vein in humans also commences at the anterior perforate substance by merging of the anterior cerebral, middle cerebral and striate veins [31]. The basal vein of each side takes its course around the midbrain and connects to the ICV or GCV [32]. The RV is the major vein of the ventral cerebral system and drains the caudo-ventral part of the hemisphere. The vein is rarely mentioned in the veterinary anatomy and has not been described in humans. BCV and RV connected with the DPS as opposed to the situation in humans, in which the basal vein empties into the ICV or GCV.

### Anastomoses of the Ventral Sinus System

The CS starts at the foramen orbitorotundum in sheep [33], but at the orbital fissure in dogs [34]. It ends near the CIS. In humans, the CS extends from the superior orbital fissure to the top of the petrous pyramid [18]. The rostral intercavernous sinus is usually absent in sheep [27]. We could not detect a rostral intercavernous sinus, neither in corrosion casts nor *in vivo* by contrast-enhanced MRV or CTV, but found a prominent CIS in all specimens. In contrast to humans [18], however, no evidence of a completely ‘circular sinus’ was found in sheep. The rostral epidural rete mirabile is situated in close vicinity to the dura mater and invaginated into the CS [35], [36]. The physiological function of this formation is thought to be the chilling of arterial blood supply to the brain. The absence of a rostral intercavernous sinus results in a horseshoe-shaped conformation of the ventral sinus system in dogs and sheep [27]. However, our data provide the first evidence for a strong anastomosis between the CS and PP, unveiling an additional outflow path from the CS. In our study, no evidence of a basilar sinus was found and the connection with the ventral internal vertebral plexus was established through the VOS.

### Course of the Emissary Veins of the Temporal Sinus and Formation of the Anterior Condylar Confluent

In numerous species, the TrS divides into the TeS and the SiS on either side, with the intraosseous course of the TeS and the SiS only be visualized in MRV. The TeS connects with the MV via the emissary vein of the retroarticular foramen in sheep [37] and other species. The present work documents two possible venous routes in Merino sheep. The first is an extracranial drainage from the TeS to the MV through the main retroarticular foramen, similar to the situation reported in dogs. The second is a strong vein connecting the TeS with the PTV through the tributary canal [17]. An anastomosis between the TeS and the OP, described by König [17], was neither seen in corrosion casts nor in MRV nor CTV. The second emissary pattern may be limited to ruminants (and horses) featuring a tributary canal of the temporal meatus of the TeS which is not observed in other species. Another novel finding in our study was a conspicuous, plexiform structure located extracranially near the openings of the jugular foramen and hypoglossal canal. To date, this venous confluent has only been reported in humans as the ‘anterior condylar confluent’ (ACC) [38], [39]. It constitutes a vascular crossroad between the intracranial venous sinuses of the caudal cranial fossa and the caudal cervical outflow tracks. The size of the human ACC ranges from 3 to 5 mm in a longitudinal direction and amounts to approximately 2 mm in its ventro-dorsal extension as seen in MRV.

### Anatomical Implications for Translational Cerebrovascular Research

The anatomical similarity between sheep and humans might provide a solid basis for translational research on cerebrovascular diseases of the brain using ovine models. Notwithstanding, some fundamental differences to the human anatomy and hemodynamic physiology need to be considered carefully. For example, encephalic drainage preferentially occurs through vertebral, but not jugular veins in a physiological prone/upright position in both sheep and man [39]. In most quadrupeds including sheep, the longitudinal axes of skull and spine as well as those of major veins in this area meet at an obtuse angle. This is in contrast to humans where the longitudinal axes of skull and spine and major veins meet at a right angle. When lying in a supine position, main venous outflow shifts to the anterior, jugular tracks. This is a physiological situation in humans and, due to the right angle in which the venous vessels meet, does not cause any drainage problems. However, when animals are placed in a non-physiological supine position (e.g. for surgery or imaging procedures in large bore clinical scanners) and/or the head is fixed in a reclined position, both jugular and vertebral veins may become stretched and kinked, severely reducing the venous drainage capacity. This may lead to a significant backlog of venous blood and a concomitant increase of the intracranial pressure, severely damaging the brain over time.

## Conclusions

Our study revealed novel aspects of the venous angioarchitecture of ovine intracranial venous sinuses and veins by means of *ex vivo* vascular corrosion casts, MRV and CTV. The detailed anatomical information obtained approves the notion of sheep as a relevant model species for translational research focussed on the cerebrovascular system. It also provides important implications for animal handling during such studies. An interspecies comparison between sheep, dogs and rats suggests that the cerebral venous angioarchitecture in large animals is better comparable with the human anatomy although substantial differences remain.

## Supporting Information

Figure S1 **Overview of the venous angioarchitecture of the sheep.** This schematic representation summarizes the intracranial sinuses and veins including their interconnections and the connections with the extracranial venous system as shown in the figures. The marked veins in the red boxes have been described for the first time in the sheep. Uppercase numbers refer to references in the main document.
(TIF)

Table S1 **Abbrevations of the described sinuses and veins according to the Nomina Anatomica Veterinaria, the Nomina Anatomica and the Terminologia Anatomica.**
(DOC)
