# Supplementary figures and images for: The Ovine Cerebral Venous System: Comparative Anatomy, Visualization, and Implications for Translational Research
Source: PLoS One. 2014 Apr 15;9(4):e92990. doi: 10.1371/journal.pone.0092990 (PMC3988027; doi:10.1371/journal.pone.0092990)

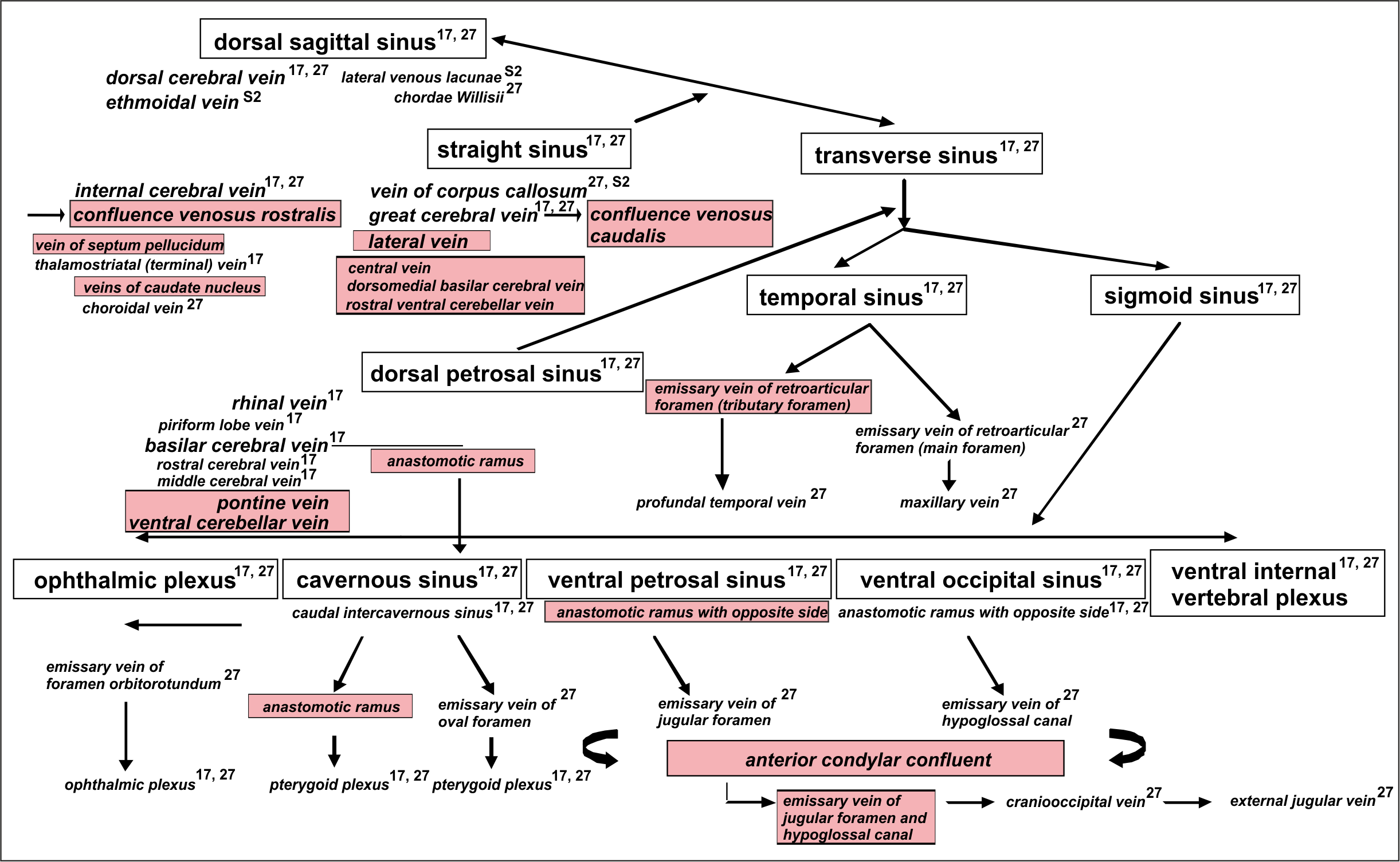

Supplement: Figure S1 — Overview of the venous angioarchitecture of the sheep. This schematic representation summarizes the intracranial sinuses and veins including their interconnections and the connections with the extracranial venous system as shown in the figures. The marked veins in the red boxes have been described for the first time in the sheep. Uppercase numbers refer to references in the main document. (TIF) [file pone.0092990.s001.tif]
