# Supplementary material for: The Ovine Cerebral Venous System: Comparative Anatomy, Visualization, and Implications for Translational Research
Source: PLoS One. 2014 Apr 15;9(4):e92990. doi: 10.1371/journal.pone.0092990 (PMC3988027; doi:10.1371/journal.pone.0092990)
Supplement: Table S1 — Abbrevations of the described sinuses and veins according to the Nomina Anatomica Veterinaria, the Nomina Anatomica and the Terminologia Anatomica. (DOC) [file pone.0092990.s002.doc]

**Table S1. Abbrevations of the described sinuses and veins according to the Nomina Anatomica Veterinaria (NAV), the Nomina Anatomica (NA) and the Terminologia Anatomica** (TA).

| **Abbr.** | **Designation** | **NAV** | **NA/TA** |
| --- | --- | --- | --- |
| **ACC** | anterior condylar confluent | - | - |
| **AR-BCV+CS** | anastomotic ramus between basilar cerebral vein and cavernous sinus | Vv. cerebri ventrales | - |
| **AR-CS+PP** | anastomotic ramus between cavernous sinus and pterygoid plexus | - | - |
| **AR-VOS** | anastomotic ramus between both ventral occipital sinuses | - | - |
| **AR-VPS** | anastomotic ramus between both ventral petrosal sinus | - | - |
| **AR-VV** | anastomotic ramus of vertebral vein | - | - |
| **BP** | basilar plexus | Sinus basilaris | Plexus basilaris |
| **BCV** | basilar cerebral vein | - | V. basalis |
| **BuV** | buccal vein | V. buccalis | V. buccalis |
| **CC** | condylar canal | Canalis condylaris | Canalis condylaris |
| **CeV** | central vein | - | - |
| **ChV** | choroidal vein | V. choroidea | V. choroidea |
| **CIS** | caudal intercavernous sinus | Sinus intercavernosus | Sinus intercavernosus posterior |
| **COV** | craniooccipital vein | - | - |
| **CS** | cavernous sinus | Sinus cavernosus | Sinus cavernosus |
| **DCV** | dorsal cerebral vein | V. cerebri dorsales | Vv. superiores cerebri |
| **DMBCV** | dorsomedial basilar cerebral vein | - | - |
| **DPS** | dorsal petrosal sinus | Sinus petrosus dorsalis | Sinus petrosus superior |
| **DSS** | dorsal sagittal sinus | Sinus sagittalis dorsalis | Sinus sagittalis dorsalis |
| **EV** | ethmoidal vein | V. ethmoidalis externa | Vv. ethmoidales |
| **EVJFHC** | emissary vein of jugular foramen and hypoglossal canal | V. emissaria foraminis jugularis | - |
|  |  | V. emissaria canalis n. hypoglossi | Plexus venosus canalis n. hypoglossi |
| **EVOrF** | emissary vein of the foramen orbitorotundum | V. emissaria foraminis orbitorotundi | - |
| **EVOvF** | emissary vein of oval foramen | V. emissaria foraminis ovalis | V. emissaria foraminis ovalis |
| **EVRF-1** | first emissary vein of retroarticular foramen (1. branch, main foramen) | V. emissaria foraminis retroarticularis | - |
| **EVRF-2** | second emissary vein of retroarticular foramen (2. branch, tributary foramen) |  | - |
| **EJV** | external jugular vein | V. jugularis externa | V. jugularis externa |
| **GCV** | great cerebral vein (Galen) | V. cerebri magna | V. magna cerebri |
| **ICV** | internal cerebral vein | Vv. cerebri internae | Vv. internae cerebri |
| **LV** | lateral vein | - | V. lateralis ventriculi lateralis |
| **LVLs** | lateral venous lacunae | Lacunae laterales | Lacunae laterales |
| **MCA** | middle cerebral artery | A. cerebri media | A. cerebri media |
| **MCV** | middle cerebral vein | - | V. media prof./supf. cerebri |
| **MV** | maxillary vein | V. maxillaris | V. maxillaris |
| **OP** | ophthalmic plexus | Plexus ophthalmicus | V. ophthalmica sup./inf. |
| **PLV** | piriform lobe vein | - | - |
| **PP** | pterygoid plexus | Plexus pterygoideus | Plexus pterygoideus |
| **PTV** | profundal temporal vein | V. temporalis profunda | Vv. temporales proff. |
| **PV** | pontine vein | - | Vv. pontis |
| **RCV** | rostral cerebral vein | - | Vv. anteriores cererbri |
| **RIS** | rostral intercavernous sinus | Sinus intercavernosus | Sinus intercavernosus anterior |
| **RV** | rhinal vein | - | - |
| **RVCrV** | rostral ventral cerebellar vein | - | Vv. superiores cerebelli |
| **SiS** | sigmoid sinus | Sinus sigmoideus | Sinus sigmoideus |
| **SS** | straight sinus | Sinus rectus | Sinus rectus |
| **STV** | superficial temporal vein | V. temporalis superficialis | Vv. temporales superficiales |
| **TeS** | temporal sinus | Sinus temporalis | Sinus temporalis |
| **TrS** | transverse sinus | Sinus transversus | Sinus transversus |
| **TSV** | thalamostriate vein | V. thalamostriata | V. thalamostriata |
| **VCC** | vein of the corpus callosum | V. corporis callosi | V. posterior/dorsalis corporis callosi |
| **VCN** | veins of caudate nucleus | - | V. nuclei caudati |
| **VCrV** | ventral cerebellar vein | Vv. cerebelli ventrales | Vv. inferiores cerebelli |
| **VOS** | ventral occipital sinus | - | Sinus occipitalis |
| **VSP** | vein of septum pellucidum | - | V. septi pellucidi |
| **VPS** | ventral petrosal sinus | Sinus petrosus ventralis | Sinus petrosus inferior |
